# Antibacterial activity of endolysin LysP70 from *Listeria monocytogenes* phage

**DOI:** 10.3389/fmicb.2025.1566041

**Published:** 2025-07-15

**Authors:** Kunzhong Zhang, Xuehui Zhao, Qing Cao, Qian Chong, Ziqiu Fan, Ji Zhi, Jiabing He, Jiayu Wang, Zhonglong Wang, Mingxia Cheng, Min Xiao, Zijian Wang, Huiwen Xue, Huitian Gou

**Affiliations:** ^1^College of Veterinary Medical, Gansu Agricultural University, Lanzhou, China; ^2^Gansu Province Animal Disease Prevention and Control Center, Lanzhou, China

**Keywords:** LysP70, antibacterial activity, *Listeria monocytogenes*, endolysin, bacteriophage

## Abstract

Endolysins, which are potential antimicrobial agents, can directly lyse gram-positive bacteria from the exterior. In this study, the endolysin gene derived from Listeria phage P70 was cloned, expressed, and purified, and designated LysP70. The antibacterial efficacy of LysP70 was comprehensively assessed through plate counting and electron microscopy. The findings indicate that LysP70 is composed of 315 amino acids and has a molecular weight of 34.2 kDa, structural stability, and peptidase activity. Successfully expressed and purified LysP70 demonstrated lytic activity against *L. monocytogenes*, but not against *Staphylococcus* or *Salmonella*. LysP70 displayed stable enzymatic activity across a range of pH levels, temperatures, and metal ion concentrations. Furthermore, LysP70 significantly inhibited *L. monocytogenes* biofilm formation and scavenged existing biofilms, while affecting the transcriptional levels of genes associated with biofilm formation. In terms of food applications, LysP70 was effective in reducing the *L. monocytogenes* count in milk by 1.9 Log_10_ CFU/mL. This study offers a novel strategy for the prevention and control of *L. monocytogenes* infection, and establishes a theoretical basis for the development of endolysin antimicrobial agents.

## 1 Introduction

*Listeria monocytogenes* is a gram-positive, foodborne pathogen that primarily affects immunocompromised individuals. Severe infections may result in sepsis, meningitis, or abortion, with a mortality rate of 20–30% (Thakur et al., 2018; Yu et al., 2019). *L. monocytogenes* is widely distributed in soil, water, decaying plant matter, and animal foodstuffs (Du et al., 2018). Remarkably, *L. monocytogenes* survives under harsh conditions such as low temperatures, high osmotic pressure, and acidic environments. During food processing, bacteria adhere to both biotic and abiotic surfaces, making it difficult to eliminate biofilms, which provide protective advantages (Qiao et al., 2022; Yu et al., 2020). Biofilms pose a substantial threat to both the food industry and public health (Fang et al., 2020). Consequently, novel strategies are urgently needed to prevent and control *L. monocytogenes*. In recent years, the broad-spectrum antibacterial activity of endolysins has attracted increasing attention (Gondil et al., 2020). Compared to bacteriophages, endolysins offer advantages such as non-replication, ease of targeted drug delivery, broad host range, and reduced drug resistance (Abdelrahman et al., 2023). They degrade the cell wall from within, facilitating the release of progeny phages (Lu et al., 2021). As cell-wall hydrolases, they play a pivotal role during the late stages of phage infection and selectively and rapidly kill bacteria (Gerstmans et al., 2018). The lyase Abtn-4 from *Acinetobacter baumannii* eliminates bacteria within 2 h and demonstrates broad-spectrum antibacterial activity against both gram-positive and gram-negative bacteria, including *Staphylococcus aureus*, *Pseudomonas aeruginosa*, *Klebsiella pneumoniae*, and *Salmonella* (Yuan et al., 2021). CHAPk, a truncated derivative of the staphylococcal phage lysin LysK, effectively prevents and disrupts staphylococcal biofilms (Arroyo-Moreno et al., 2021). Endolysins can be used independently, or in combination with other antimicrobial agents, to achieve synergistic antibacterial effects. The endolysin Cpl-711 has demonstrated synergistic activity with various antibiotics against multidrug-resistant *Streptococcus pneumoniae* strains (Letrado et al., 2018). When combined with polymyxin, LysMK34 reduced the minimum inhibitory concentration of polymyxin 32-fold (Abdelkader et al., 2020; Abdelkader et al., 2022). Furthermore, endolysins have been extensively used for disease treatment and in the food industry. *S. aureus* LysSYL has demonstrated efficacy in treating peritonitis in BALB/c mice by significantly enhancing survival rates and reducing the bacterial load across multiple tissues significantly (Liu et al., 2024). In a mouse skin infection model, the application of PlyKp104 reduced the bacterial counts in the skin by approximately 2 log units in *K. pneumoniae*-infected mice (Euler et al., 2023). The endolysin rLysJNwz, in combination with EDTA, reduced *Salmonella* contamination in eggs and lettuce by 86.7 and 86.5%, respectively (Shen et al., 2023). A high concentration of LysCP28 (500 μg/mL) completely eradicated *Clostridium perfringens* in ducks at 4°C over a 24 h period (Lu et al., 2023). These findings highlight the potential use of phage-derived lysins as novel antimicrobial agents.

Currently, research on the antimicrobial effects and biofilm disruption by phage endolysins targeting *L. monocytogenes* remains limited. In this study we report the acquisition of LysP70 endolysin through prokaryotic expression and subsequent purification. We further assessed the lytic potency of LysP70 against *L. monocytogenes*, its effect on bacterial biofilms, and its potential applications in milk and lettuce.

## 2 Materials and methods

### 2.1 Bacterial strains

*Listeria monocytogenes*, *Listeria ivanovill*, *Listeria welshimeri*, *Listeria innocua*, *Staphylococcus* and *Salmonella*, were obtained from the Veterinary Public Health Laboratory of Gansu Agricultural University. Competent *Escherichia coli* BL21(DE3) cells were obtained from Beijing Qingke Biotechnology Co. (Beijing, China).

### 2.2 Reagents and primers

The plasmid DNA extraction kit was procured from Beijing Jinsha Biotechnology Co., protein markers were sourced from Shenggong Bioengineering (Shanghai, China) Co, and the Ni-NTA His-tag protein purification resin was obtained from GE Healthcare (United States). The Total RNA Isolation Kit, HiScript II Q RT SuperMix, and SYBR qPCR Master Mix were procured from Nanjing Nuoweizan Biotechnology Co. (China). IPTG was obtained from Beijing Solaibao Technology Co. SYTO9 and propidium iodide (PI) were purchased from Shaanxi Xinyan Bomei Biotechnology Co. (China), Primers were synthesized by Beijing Qingke Biotechnology Co. The primer sequences were as follows: T7, 5′-TAATACGACTCACTATAGGG-3′; T7 ter, 5′-GCTAGTTATTGCTCAGCGG-3′.

### 2.3 Bioinformatics analysis of LysP70

The gene sequence of *Listeria* bacteriophage P70 endolysin (GenBank accession number: NC_018831.1) was downloaded from the National Center for Biotechnology Information database and designated LysP70. Online tools^1^ were used to analyze the molecular weights and isoelectric points of the amino acid sequences. Conserved domain analysis was performed using the Conserved Domains Database.^2^ The LysP70 tertiary structure was predicted using Swiss-Model.^3^

### 2.4 Construction and identification of the pET28a-LysP70 expression vector

*LysP70* was synthesized by Shengong Bioengineering (Shanghai) Co. and the recombinant plasmid pET28a-LysP70 was constructed. The recombinant plasmid was transformed into *E. coli* Top10 competent cells, inoculated with kanamycin (50 μg/mL), and incubated overnight at 37°C. Single colonies were randomly selected for polymerase chain reaction (PCR) verification using the universal primers T7 and T7 ter.

### 2.5 Expression and purification of recombinant protein

The recombinant plasmid was transformed into competent *E. coli* BL21(DE3) cells. Single colonies were selected and cultured in Luria Bertani broth containing kanamycin until an OD_600nm_ of 0.6 was reached. Protein expression was induced by adding IPTG (0.5 mM), followed by incubation at 16°C overnight. The cells were harvested by centrifugation at 13,000 × *g* for 15 min at 4°C, washed with phosphate-buffered saline (PBS), resuspended, and lysed by sonication on ice. The lysate was centrifuged at 13,000 × *g* for 20 min at 4°C, and the supernatant was collected. The recombinant protein was purified using an Ni-NTA affinity chromatography column and analyzed using sodium dodecyl-sulfate polyacrylamide gel electrophoresis (SDS-PAGE). The buffer was replaced with PBS in an ultrafiltration centrifuge tube, and the protein concentration was determined using a BCA Protein Assay Kit, according to the manufacturer’s instructions.

### 2.6 Determination of lytic activity of LysP70

*Listeria monocytogenes* Li4 cells in the logarithmic growth phase were inoculated into 1.2% brain heart infusion (BHI) medium. After decanting the plates, 8 μL of endolysin LysP70 was added dropwise to the medium surface, whereas the control received an equivalent volume of PBS. The plates were then incubated at 37°C for 8 h, and the lysis plaque were observed.

A bacterial suspension, adjusted to a concentration of 10^8^ CFU/mL with PBS, was mixed with varying concentrations of LysP70 (400, 200, 100, and 50 μg/mL). The control group was administered PBS. The OD_600nm_ was measured after 2 h of incubation at 37°C with shaking.

The bacterial suspension (100 μL) was then mixed with various concentrations of LysP70. The mixtures were incubated at 37°C with shaking for 2 h, and samples were collected every 20 min for dilution and plating. The plates were incubated at 37°C for 24 h before colony counting.

### 2.7 Evaluation of LysP70 lysis spectrum

Twenty-three strains of *L. monocytogenes*, one strain each of *L. ivanovill*, *L. welshimeri*, and *L. innocua*, *Salmonella* strains, and *Staphylococcus* were chosen for the evaluation of the LysP70 lysis spectrum, using the same methodology as described in Section 2.6.

### 2.8 Assessment of LysP70 stability

#### 2.8.1 Effects of pH on LysP70 lytic activity

Li4 cells were cultured until the logarithmic phase (OD_600nm_ = 0.6–0.8) was reached, followed by centrifugation of the bacterial suspension at 7,000 × *g* for 5 min. The supernatant was discarded and the bacterial pellet was washed three times with sterile PBS. Subsequently, the bacterial pellets were resuspended in PBS at different pH values. In the experimental group, 100 μL of bacterial suspension was supplemented with LysP70 at a final concentration of 400 μg/mL. In the control group, an equivalent volume of PBS was added in lieu of LysP70. Both groups were incubated at a constant temperature of 37°C for 2 h. Subsequently, 100 μL of the mixture was removed for tenfold serial dilution, plated, and colony counting was performed after 24 h incubation at 37°C.

#### 2.8.2 Effects of temperature on LysP70 lytic activity

Li4 cells were cultured until the logarithmic phase was reached, centrifuged for 5 min, and the bacterial pellet was washed three times with PBS, and then resuspended with PBS. The protein was incubated in water at various temperatures (20, 30, 37, 40, 50, 60, 70, 80, and 90°C) for 30 min and then mixed with 100 μL of bacterial suspension after being returned to room temperature. The final concentration of LysP70 was adjusted to 400 μg/mL, whereas the control group was supplemented with an equal volume of PBS. The mixture was incubated at 37°C for 2 h, followed by removal of 100 μL for tenfold serial dilution, plating, and colony counting after 24 h incubation at 37°C in an inverted position.

#### 2.8.3 Effects of Na^+^ and Zn^2+^ on LysP70 lytic activity

The bacterial suspension was prepared according to the procedure outlined in Section 2.8.2. Purified LysP70 was introduced into the prepared 100 μL bacterial suspension, followed by the addition of Na^+^ and Zn^2+^ at varying concentrations. The mixture was incubated at 37°C for 2 h, followed by removal of 100 μL for tenfold serial dilution, and plated onto a BHI agar plate. Colony counting was carried out following 24 h incubation at 37°C.

### 2.9 Effect of LysP70 on *Listeria monocytogenes* biofilm formation

#### 2.9.1 Inhibition of biofilm formation by LysP70

A bacterial solution at a concentration of 10^6^ CFU/mL was mixed with an equal volume of LysP70 and added to 96-well and 12-well plates that had been seeded with cell crawlers. All plates were incubated at 37°C for 24 and 72 h. Subsequent procedures followed the crystal violet staining method described by Bolocan et al. (2017).

The fluorescence staining method described previously (Pennone et al., 2019) was used. Briefly, the cell crawlers from the 12-well plates were removed, washed with sterile PBS, fixed in 2.5% glutaraldehyde for 20 min, stained with STYO9/PI staining solution for 20 min, then the staining solution was discarded and washed again with PBS. After air-drying, the samples were sealed with a fluorescent burst sealer and observed under a fluorescence microscope.

Scanning electron microscopy (SEM) was performed as previously described (Wang et al., 2023). Briefly, the prepared crawler slices were fixed in 2.5% glutaraldehyde, rinsed three times with PBS, and post-fixed with 1% osmium acid at room temperature in the dark for 2 h. The samples were subjected to a graded ethanol dehydration series (30, 50, 70, 80, 90, 95, and 100%), followed by critical point drying and gold sputtering and then examined using SEM.

#### 2.9.2 Degradation of biofilm by LysP70

Bacterial suspensions in 96-well plates were incubated at 37°C for 24 and 72 h, after which the supernatant was aspirated. Biofilm-containing wells in the 96-well plate were treated with 400 μg/mL LysP70 and incubate at 37°C for 2 h to treat the formed biofilm. The efficacy of LysP70 in degrading biofilms was ascertained using the three methodologies outlined in Section 2.9.1.

### 2.10 Effect of LysP70 on the expression of *Listeria monocytogenes* biofilm-related genes

Li4 cells were cultured at 37°C for 24 h and the bacterial concentration was adjusted to 10^5^ CFU/mL. The diluted bacterial suspension was treated with LysP70. Following incubation at 37°C for 24 and 72 h, total RNA was extracted from the bacterial cultures using an RNA extraction kit, and cDNA was synthesized by reverse transcriptase HiScript II Q RT SuperMix.

The genes *gyrB*, *agrA*, *luxS*, *lmo2504*, *degU*, *flip*, *agrB*, *sigB*, *motB*, *flgE*, *actA* were chosen as targets to detect expression. *gyrB* was used as a reference gene. Real-time quantitative PCR was performed using a SYBR qPCR Master Mix kit for fluorescence detection. The PCR mixture contained 10 μL of SYBR qPCR Master Mix, 0.4 μL of both upstream and downstream primers (10 μM), 0.4 μL of 50 × ROX Reference Dye 1, 1 μL of cDNA, and ddH_2_O in a final volume of 20 μL. The thermal cycling conditions were as follows: 95°C for 30 s, followed by 95°C for 10 s and 60°C for 30 s, for a total of 40 cycles. The primer sequences are listed in Table 1. Gene expression levels were quantified using the 2^-ΔΔCt^ method.

**Table 1:** Primer sequence of RT-qPCR.

Gene	Primer sequence (5′–3′)
*gyrB*	F: GAGTTGGTGCATCGGTAGTT
	R: TCGCCTTGTTCTTCCATATCC
*agrA*	F: GATCCGCGTGGTTTCATTATTT
	R: TCAAGCGCTTCCACCTTATAC
*luxS*	F: GGCGTCCATGGAGATGAAATA
	R: CAAGTTCTGCCATCAAATGTTCT
*lmo2504*	F: ACAGCGACAGGGATTGATTTA
	R: CTGCAATTGCTTGTCCGTATC
*degU*	F: CGCCATTTAGCAAGCACTAAC
	R: GCAATACCTCGCACTCTCTATG
*flip*	F: TGTTGGACTGGCACTGTTT
	R: GATGCGCTCCACTCTTCTTT
*agrB*	F: CGAAAGACCGCTGGAAAGA
	R: CCGTATACGAGAGCAAACTTCA
*sigB*	F: CGCGCCGAATCAAAGAATTAG
	R: TCCGTGACACCGATGAAATC
*motB*	F: CAGTCGGGAAGTGCAGAATTA
	R: ACAATCCCTTCCATCGTTCC
*flgE*	F: CCACAGTAAACGGCAAACAAG
	R: CCGTCAGAAGTTGGTGAGAAT
*actA*	F: CAGCGAGACAACAGAAGAAGA
	R: GGAACCGGACTGCTAGTAAAC

### 2.11 Lytic activity of LysP70 in milk

Individual colonies of Li4 were carefully selected from PALCAM agar plates, cultured to the logarithmic growth phase, thoroughly washed with PBS, and resuspended. The resuspended culture was inoculated into milk at a dilution of 1:100, resulting in a final concentration of 10^6^ CFU/mL. Endolysin was introduced into milk samples at concentrations of 400 and 200 μg/mL. After incubation for 0, 2, 4, 6, 8, 12, 24, and 48 h, 100 μL aliquots of the milk samples were collected, serially diluted, plated onto BHI agar, and the colonies were enumerated after 24 h incubation at 37°C.

### 2.12 Lytic activity of LysP70 in lettuce

Fresh lettuce was thoroughly rinsed with distilled water, sectioned into 1 cm^2^ squares, deposited into sterile Petri dishes, and subjected to UV irradiation for 30 min to achieve sterility. The lettuce pieces were inoculated with 100 μL of Li4 bacterial suspension at a concentration of 10^8^ CFU/cm^2^ after air-drying for 15–20 min. Distribute the endolysin LysP70 (100 μL) at concentrations of 400 and 200 μg/mL to the surface of lettuce leaves, followed by gentle leaf flipping with sterile forceps to ensure uniform endolysin coverage across the lettuce interface. Whereas the control group received an equivalent volume of PBS buffer, and both were incubated at 4°C and 30°C. At intervals of 0, 2, 4, 6, 8, 12, 24, and 48 h, the lettuce samples were excised and immersed in sterile test tubes containing 1 mL of PBS, followed by homogenization. One hundred microliters of the homogenate was serially diluted tenfold with sterile PBS, plated onto BHI agar, and incubated at 37°C for 24 h, after which the bacterial count was determined.

### 2.13 Data analysis

Data visualization and analysis were performed using GraphPad Prism 8.0 and Excel 2016. Statistical significance was assessed using *t*-tests and one-way/multivariate analysis of variance. Statistical significance was interpreted as follows: no asterisk indicates no significant difference (*p* > 0.05), a single asterisk indicates a significant difference (*p* < 0.05), and double or triple asterisks indicate highly significant differences (*p* < 0.01 and *p* < 0.001, respectively).

## 3 Results and analysis

### 3.1 Bioinformatics and SDS-PAGE analysis of LysP70

**Figure 1:**
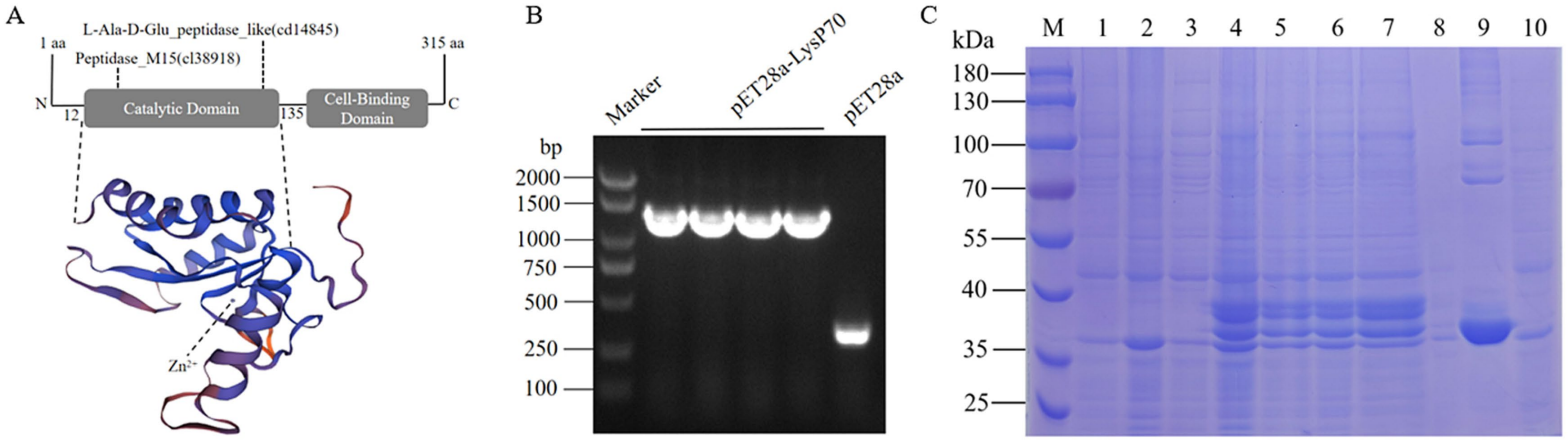
Characterization and purification of LysP70. **(A)** Schematic illustration of the structure of LysP70. The upper part is the schematic diagram of the conservative domain, and the lower part is the three-level structure model. **(B)** PCR identification of the recombinant plasmid pET28a-LysP70. **(C)** SDS-PAGE analysis of the recombinant protein LysP70. M: Marker. 1: Uninduced pET28a. 2: Induced pET28a. 3: Uninduced pET28a-LysP70. 4: Induced pET28a-LysP70. 5: Whole protein before fragmentation. 6: Whole protein after fragmentation. 7: Supernatant. 8: Precipitate. 9: Purified protein. 10: Flow through.

The LysP70 protein consists of 315 amino acids, with a molecular weight of 34.2 kDa, a theoretical isoelectric point of 9.86 (Appendix 1). Protein domain analysis revealed that LysP70 contains five active sites and three Zn^2+^-binding sites (Appendix 2). LysP70 is comprised of two distinct domains: the N-terminal catalytic domain (CD), which is responsible for the cleavage of L-Ala and D-Glu residues in the cell wall peptidoglycan, and the conserved L-Ala-D-Glu domain. The peptidase-like domain is classified within the Peptidase_M15 superfamily, with predicted sites spanning amino acids 12–135. The C-terminal region contains a novel cell wall-binding domain (CBD) that mediates attachment to the bacterial cell wall. The predicted tertiary structure of LysP70 is shown in Figure 1A.

To construct the recombinant plasmid pET28a-LysP70, four randomly selected monoclonal bacterial strains were screened using PCR. As shown in Figure 1B, amplification of a 1,300 bp gene fragment confirmed the successful construction of the plasmid. Following successful transformation of the recombinant plasmid pET28a-LysP70 into *E. coli* BL21(DE3), protein expression was induced using IPTG, followed by purification. Protein bands were observed at approximately 35 kDa, with a molecular weight consistent with the predicted size, confirming successful purification of LysP70 (Figure 1C).

### 3.2 Determination of LysP70 lytic activity

**Figure 2:**
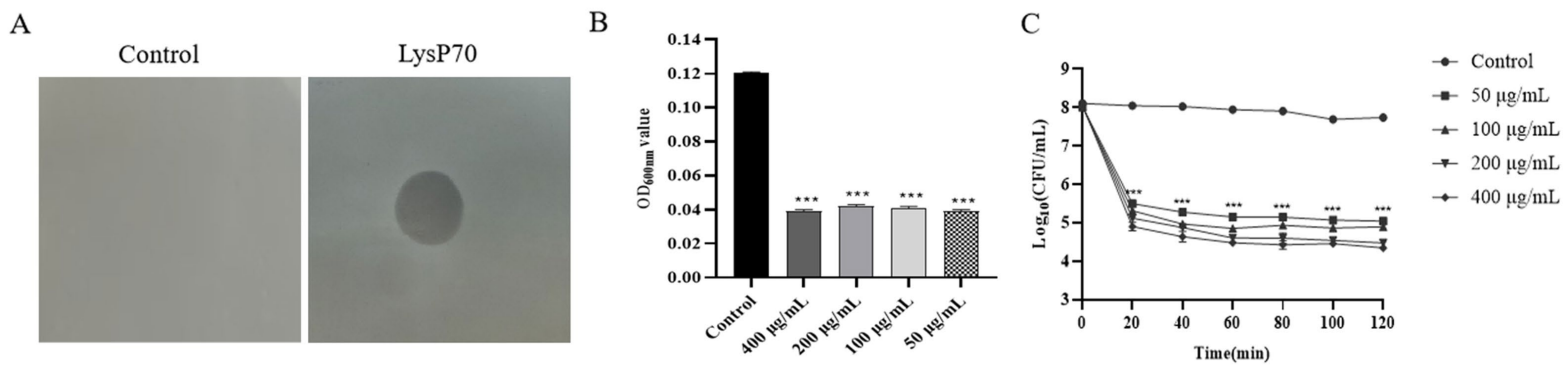
Determination of lytic activity of LysP70. **(A)** Lytic activity of LysP70 on agar. Obvious lysis plaque appeared on the plates with 8 μL LysP70 intravenous drip. **(B)** Lytic activity of LysP70 in liquid environment. The determined OD_600nm_ value represents the concentration of the bacterial solution. After the action of LysP70, the OD_600nm_ value decreased significantly and the concentration of the bacterial solution decreased. **(C)** Bactericidal activity of LysP70 in vitro. LysP70 exhibited strong lytic activity against *L. monocytogenes* after 20 min of treatment.

Compared to the control group, the LysP70-treated group exhibited a clear lysis plaque, indicating that purified LysP70 effectively lysed *L. monocytogenes* strain Li4, demonstrating significant lytic activity (Figure 2A). The LysP70 concentration ranged from 50 to 400 μg/mL, and the bacterial solution became clear and transparent (Appendix 3), accompanied by a significant decrease in OD_600nm_ values compared to the control group (*p* < 0.001; Figure 2B). When the LysP70 concentration was 400 μg/mL, the number of viable bacteria was reduced by approximately 3.4 Log_10_ CFU/mL (*p* < 0.001). At 50 μg/mL, the number of viable bacteria was reduced by approximately 2.7 Log_10_ CFU/mL (*p* < 0.001), further indicating the strong bactericidal effect of LysP70. Moreover, the number of viable bacteria significantly decreased after 20 min of incubation with LysP70 (Figure 2C).

### 3.3 Determination of LysP70 lysis spectrum

As shown in Table 2, LysP70 demonstrated broad lytic activity against multiple *Listeria* species, including *L. monocytogenes*, *L. ivanovii*, *L. welshimeri*, and *L. innocua* but no lytic activity against *Staphylococcus* or *Salmonella*. These results indicate that LysP70 exhibits broad lytic activity against *Listeria* species, but does not lyse *Staphylococcus* or *Salmonella*.

**Table 2:** Results of the lysis spectrum determination of LysP70.

Strain type	Name	Serotype	Lysis plaque	Strain type	Name	Serotype	Lysis plaque
*Listeria monocytogenes*	164–5	1/2a	+	*Listeria monocytogenes*	Li2	1/2a	+
	A77-1-2	1/2a	+		Li1	1/2b	+
	Li3	1/2a	+		Li7	1/2b	+
	D46-1-1	1/2a	+		Li4	1/2b	+
	A83-2-1	1/2c	+		D40	1/2c	+
	2–1	1/2a	+		C64-2	1/2c	+
	83–1	1/2a	+	*Listeria ivanovill*			+
	149–1	1/2a	+	*Listeria welshimeri*			+
	6–56–1-2	1/2c	+	*Listeria innocua*			+
	A85-1-1	1/2b	+	*Staphylococcus albus*			−
	ATCC19115	4b	+	*Staphylococcus citreus*			−
	5–90–1-2	1/2c	+	*Salmonella typhimurium*	N34-1		−
	1–12–2-2	1/2c	+		N14-1		−
	2–20-1	4b	+	*Salmonella typhi*	16–1		−
	CVCC1598	4b	+		D04-1		−
	Li6	1/2a	+	*Salmonella choleraesuis*	T12-2		−
	SJ14	1/2c	+		K49-1		−

“+” represents a lysis plaque; “-” indicates absence of a lysis plaque.

### 3.4 Determination of the stability of LysP70

**Figure 3:**
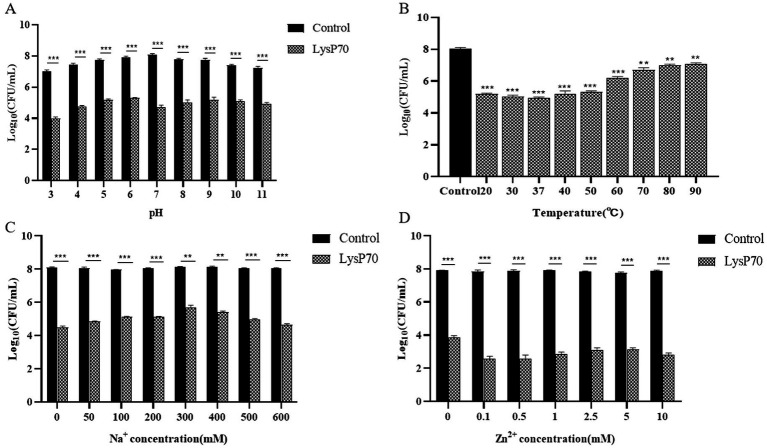
Determination of the stability of LysP70. **(A)** Effect of pH on LysP70 lytic activity. **(B)** Effect of temperature on LysP70 lytic activity. **(C)** Effect of Na^+^ on LysP70 lytic activity. **(D)** Effect of Zn^2+^ on LysP70 lytic activity. The data are expressed as the mean ± SD of three independent experiments. Statistical significance was determined by multifactorial analysis of variance, **p < 0.01, *** *p* < 0.001.

LysP70 exhibited high enzymatic activity within a pH range of 3–11(Figure 3A), demonstrating its resistance to both acidic and alkaline conditions. At pH 7, LysP70 demonstrated relatively high lytic activity, with a reduction of approximately 3.3 Log_10_ CFU/mL in viable bacteria (*p* < 0.001) compared to the control group, suggesting that pH 7 is the optimal pH value for LysP70. In the temperature stability assay (Figure 3B), LysP70 displayed high activity within a temperature range of 20–50°C. At temperatures ranging from 60°C to 90°C, LysP70 activity was significantly reduced, yet it retained some activity, indicating its ability to withstand high temperatures. The optimal temperature for LysP_70_ activity was 37°C.

In the Na^+^ stability assay (Figure 3C), without Na^+^, the number of viable bacteria decreased by approximately 3.6 Log_10_ CFU/mL. After the addition of 300 mM Na^+^, the number of viable bacteria decreased by approximately 2.5 Log_10_ CFU/mL, suggesting that Na^+^ inhibited the enzyme activity of LysP70, although LysP70 retained its activity compared to the control group (*p* < 0.001). As the Na^+^ concentration increased from 50 to 300 mM, the inhibitory effect strengthened. However, between 300 and 600 mM, the inhibitory effect diminished as the Na^+^ concentration increased. In the Zn^2+^ stability assay (Figure 3D), without Zn^2+^, the number of viable bacteria decreased by approximately 4.1 Log_10_ CFU/mL. At 0.5 mM, the number of viable bacteria decreased by approximately 5.3 Log_10_ CFU/mL, indicating that Zn^2+^ enhanced the enzymatic activity of LysP70. The addition of Zn^2+^ from 0.1 to 10 mM promoted the enzymatic activity of LysP70, although the effect was irregular.

### 3.5 Effects of LysP70 on *Listeria monocytogenes* biofilm

#### 3.5.1 Inhibitory effect of LysP70 on biofilm

**Figure 4:**
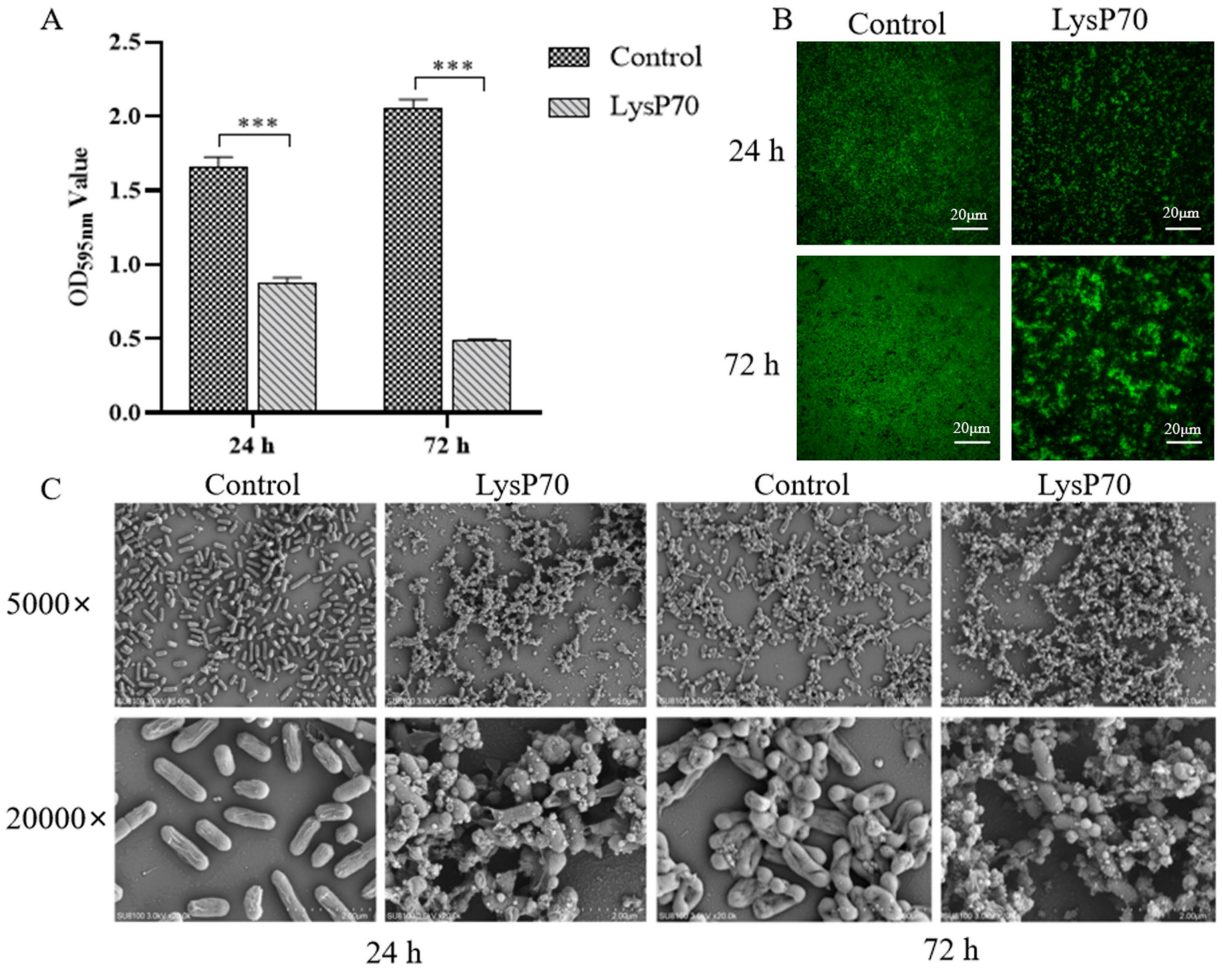
Inhibitory effect of LysP70 on *L. monocytogenes* biofilm. **(A)** Crystal violet staining. The 400 μg/mL LysP70 was co-cultured with *L. monocytogenes* for 24 h and 72 h. PBS was used as the control group, and the OD_595nm_ values of the two groups were measured to represent the amount of biofilm. **(B)** Fluorescent staining. Weak green fluorescence in the LysP70-treated group indicates a reduction in viable *L. monocytogenes* cells. **(C)** Scanning Electro Microscopy (SEM). Changes in bacterial morphology and abundance are shown at magnifications of 5,000 × and 20,000 × .

Crystal violet staining revealed a significantly reduced OD_595nm_ value in the biofilm compared to that in the control group (*p* < 0.001; Figure 4A). Fluorescence staining demonstrated that the number of viable bacteria in the LysP70 treatment group was lower than that in the control group, and the structure was more dispersed (Figure 4B). SEM revealed that the bacterial morphology in the control group was intact with well-defined edges, whereas the morphology of the bacteria treated with LysP70 was disrupted and deformed (Figure 4C). These results strongly suggest that LysP70 effectively inhibits biofilm formation by disrupting the biofilm structure, reducing biomass, and compromising bacterial morphology.

#### 3.5.2 Degradation of biofilm by LysP70

**Figure 5:**
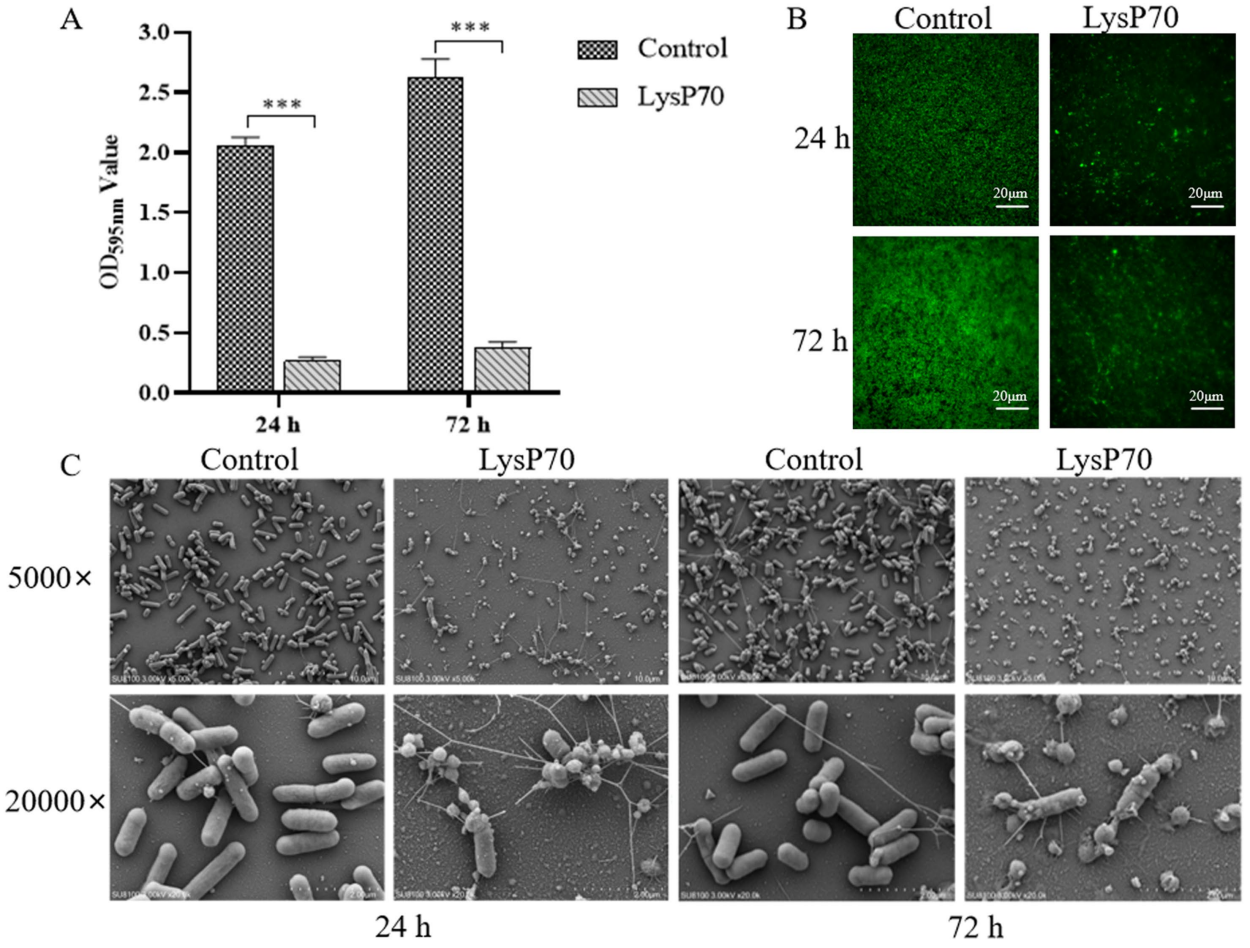
Scavenging effect of LysP70 on *L. monocytogenes* biofilm. **(A)** Crystal violet staining. *L. monocytogenes* biofilms were cultured for 24 h and 72 h, followed by treatment with 400 μg/mL LysP70 for 2 h, with PBS as the control group. The OD_595nm_ values of both groups were measured to quantify biofilm biomass. **(B)** Fluorescent staining. Weak green fluorescence in the LysP70-treated group indicates a reduction in viable *L. monocytogenes* cells. **(C)** Scanning Electro Microscopy (SEM). Changes in bacterial morphology and abundance are shown at magnifications of 5,000 × and 20,000 × .

To investigate the ability of LysP70 to clear preformed biofilms, biofilms were cultured for 24 and 72 h before undergoing a 2 h treatment with LysP70. The clearance effect of LysP70 on the preformed biofilm was assessed. Crystal violet staining revealed a significantly reduced OD_595nm_ value in the biofilm compared to that in the control group (*p* < 0.001; Figure 5A). Fluorescence staining demonstrated that the biofilm in the control group exhibited a dense network structure with a substantial number of viable bacteria, whereas the biofilm structure became more dispersed, and the bacterial count decreased following treatment (Figure 5B). SEM revealed that compared to the control group, the morphology of the bacteria treated with LysP70 was disrupted and deformed (Figure 5C). These findings demonstrate that LysP70 can effectively disrupt and clear preformed biofilms, even those that have matured over extended periods.

### 3.6 Effect of LysP70 on transcription of biofilm-related genes in *Listeria monocytogenes*

**Figure 6:**
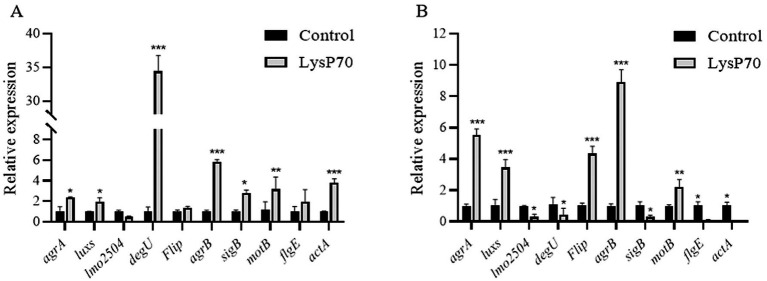
Relative expression of biofilm-related genes in *L. monocytogenes* treated with LysP70. **(A)** Analysis of relative gene expression after 24 h of LysP70 treatment. **(B)** Analysis of relative gene expression after 72 h of LysP70 treatment. The horizontal axis represents biofilm-associated genes, and the vertical axis indicates the relative expression levels of the genes. Data are presented as mean ± SD of three independent experiments. Statistical significance was determined by multifactorial analysis of variance, **p < 0.01, ***p < 0.001.

The effects of LysP70 on gene transcription were evaluated using RT-qPCR, targeting genes associated with quorum sensing, motility, virulence, cell wall binding, and stress responses. *gyrB* served as the internal reference gene. After 24 h of treatment, significant upregulation was observed in quorum sensing-related (*agrA*, *luxS*, and *agrB*), stress response (*sigB*), motility-related (*motB*), and virulence factor (*actA*) genes compared to the control. Meanwhile, *flip* and *flgE* showed slight increases (*p* > 0.05), whereas the cell wall-binding protein gene *lmo2504* exhibited a slight decrease (*p* > 0.05; Figure 6A). A time-dependent shift in gene expression was observed after 72 h of treatment. The expression levels of *agrA*, *luxS*, *flip*, *agrB*, and *motB* remained significantly upregulated, whereas those of *lmo2504*, *degU*, *sigB*, *flgE*, and *actA* significantly decreased (Figure 6B). These results indicate that LysP70 not only disrupts biofilm integrity but also modulates critical genetic pathways involved in bacterial communication, motility, and virulence. This dual action highlights its potential as a highly effective antibiofilm agent.

### 3.7 Lytic activity of LysP70 in milk

**Figure 7:**
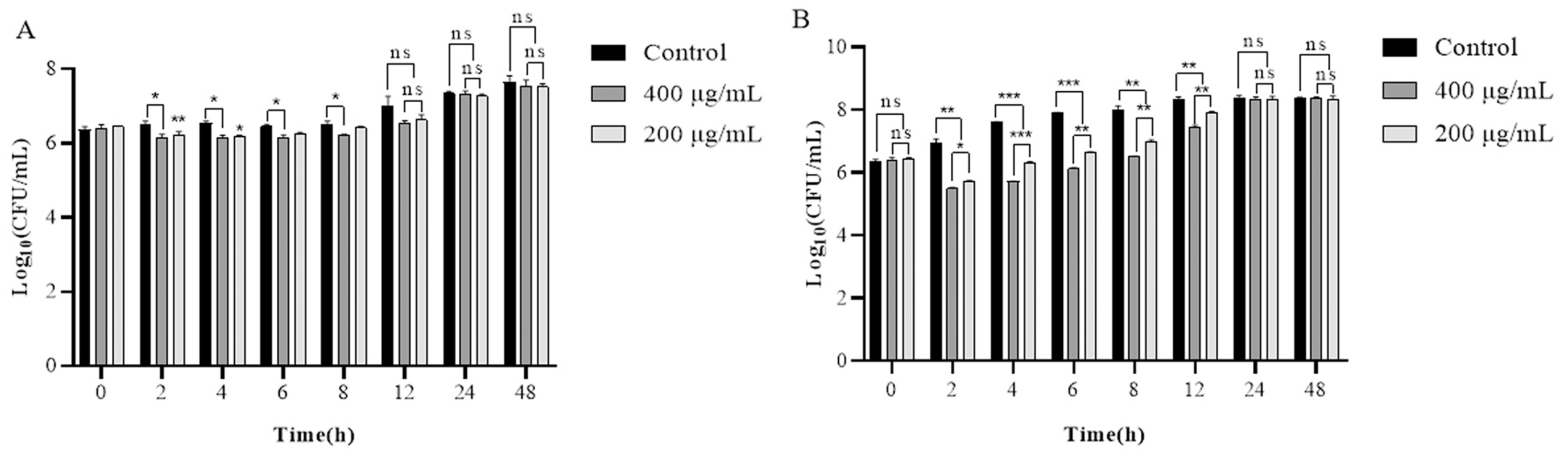
Lytic activity of LysP70 in milk. **(A)** Lytic activity of LysP70 in milk at 4°C. **(B)** Lytic activity of LysP70 in milk at 30°C. Determination of the lytic activity of LysP70 in milk under different temperatures and time conditions. The number of viable bacteria is expressed by the order of magnitude represented by Log_10_. Data are presented as mean ± SD of three independent experiments. Statistical significance was determined by multifactorial analysis of variance, **p < 0.01, ***p < 0.001.

At 4°C, over an interval of 2–12 h, the viable bacterial count in the experimental group was reduced by approximately 0.3–0.5 Log_10_ CFU/mL relative to the control group. After 24 h and 48 h, the viable bacterial count in the experimental group converged with that of the control group, with no statistically significant divergence (Figure 7A). At 30°C, the population of live bacteria in the control group exhibited a continuous increase over a 12 h period, escalating from an initial concentration of 6.3 Log_10_ CFU/mL to 8.3 Log_10_ CFU/mL. The optimal lysis activity was achieved at the 4-h incubation mark with concentrations of 400 and 200 μg/mL of LysP70, corresponding to a reduction in the viable bacterial count of 1.9 Log_10_ CFU/mL and 1.3 Log_10_ CFU/mL, respectively. After 24 and 48 h of incubation, the live bacterial count in the experimental group remained congruent with that in the control group, with no significant difference (Figure 7B). Collectively, LysP70 exhibited heightened lysis activity at 30°C as opposed to 4°C.

### 3.8 Lytic activity of LysP70 in lettuce

**Figure 8:**
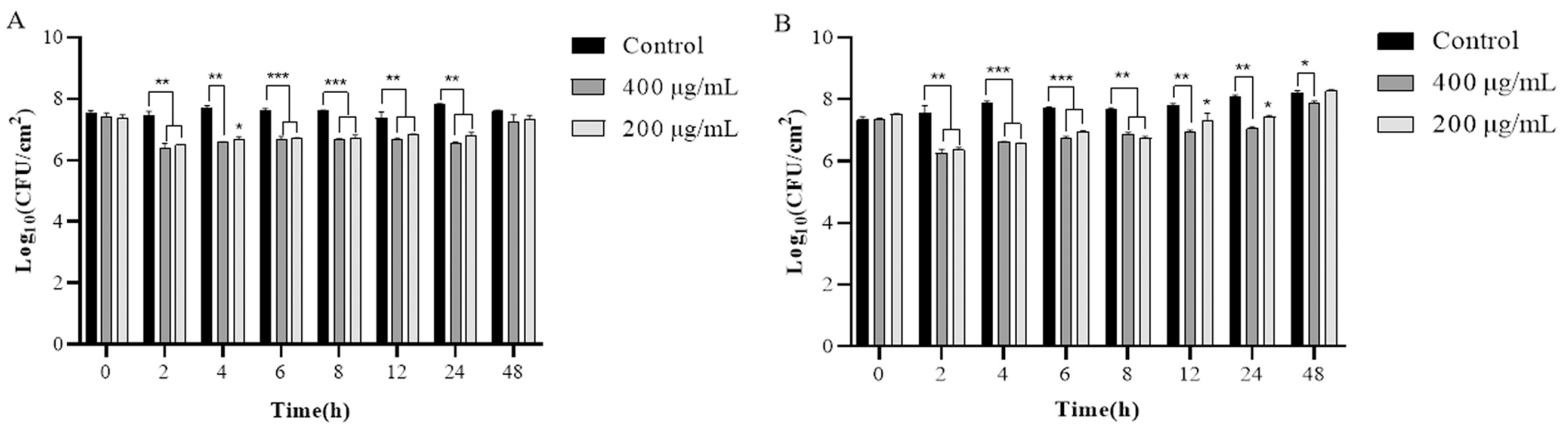
Lytic activity of LysP70 in lettuce. **(A)** Lytic activity of LysP70 in lettuce at 4°C. **(B)** Lytic activity of LysP70 in lettuce at 30°C. Determination of the lytic activity of LysP70 in lettuce under different temperatures and time conditions. The number of viable bacteria is expressed by the order of magnitude represented by Log_10_. Data are presented as mean ± SD of three independent experiments. Statistical significance was determined by multifactorial analysis of variance, **p < 0.01, ***p < 0.001.

During the 24 h incubation period of LysP70 at 4°C, the viable bacterial count in the experimental group was reduced by approximately 0.53–1.23 Log_10_ CFU/cm^2^ relative to the control group (Figure 8A). Incubation of LysP70 at 30°C for 4 h resulted in an optimal lysis effect, characterized by a reduction in viable bacterial count by 1.29 Log_10_ CFU/cm^2^ and 1.33 Log_10_ CFU/cm^2^ at concentrations of 200 and 400 μg/mL, respectively, as compared to the control group (Figure 8B). However, after 48 h at both 4°C and 30°C, no significant differences were observed between the experimental and control groups in terms of viable bacterial counts.

## 4 Discussion

Prolonged overuse of antibiotics has led to the emergence of drug-resistant bacterial strains, complicating treatment strategies and increasing public health concerns (Li et al., 2016). Consequently, the development of novel antibacterial agents is of paramount importance to prevent and control *L. monocytogenes* infections and safeguard food safety. Endolysins are a class of peptidoglycan hydrolases that target bacterial cell wall peptidoglycans, making them less prone to developing resistance, and are considered a promising alternative to antibiotics (Oechslin et al., 2021). Phage endolysins typically exhibit a modular structure, with the N-terminus serving as the CD responsible for enzymatic activity, and the C-terminus containing one or two CBDs (Liu et al., 2023). Domain analysis of LysP70 revealed that its N-terminus contains an L-Ala-D-Glu peptidase-like domain, whereas its C-terminus remains uncharacterized, suggesting a novel CBD, consistent with reports of endolysins containing uncharacterized binding domains (Kim et al., 2020). Endolysins exhibit diverse mechanisms for cleaving cell wall peptidoglycans owing to variations in their CDs and are classified into five categories: N-acetylmuramidase, N-acetylglucosaminase, transglycosylase, endopeptidase, and amidase (Love et al., 2020). LysP70, a member of the Peptidase_M15 superfamily, functions as an endopeptidase and cleaves the peptide bond between L-Ala and D-Glu in the bacterial cell wall peptidoglycan to exert its bactericidal effects. The active site of LysP70 includes three Zn^2+^-binding sites, which are characteristic of many Peptidase_M15 proteins. Zn^2+^ plays a crucial role in maintaining the protein structure and influencing folding and enzymatic function (Zhang et al., 2021).

Bacteriophage endolysins are promising antibacterial agents, and their lytic activity and spectrum are critical indicators of their antimicrobial potential. In this study, LysP70 rapidly lysed *L. monocytogenes* but showed no effect on *Staphylococcus* and *Salmonella* species. This specificity likely results from the targeting capability of the lyase CBD toward bacterial receptors, as well as differences in bacterial cell wall composition (Reith and Mayer, 2011; Yuan et al., 2012).

Recent studies have highlighted the importance of endolysin stability for its efficacy and potential applications (Haddad Kashani et al., 2018). pH and temperature stability tests demonstrated that LysP70 remained active across a broad pH range and under high-temperature conditions, exhibiting acid–base and thermal resistance. This is consistent with the observation that LysSTG2 maintains stability over a wide pH range and at high temperatures, in contrast to P9ly, which loses its activity under acidic and high-temperature conditions (Zhang et al., 2021; Wang et al., 2022). This difference may be attributed to the amino acid residues in the active site of the enzyme, as varying residues exhibit different sensitivities to pH and temperature. Metal ions are crucial for enzyme function, and many phage lyases are metal-dependent, affecting both functional domain activity and stability of their three-dimensional conformation. The effects of metal ions on LysP70 activity were also evaluated. Na^+^ was found to suppress lysis activity, whereas Zn^2+^ enhanced it, likely because of its catalytic role in stabilizing the enzyme’s structure and enhancing its activity (Rogowska et al., 2024). Notably, LysP70 retained its bactericidal function, even in the absence of metal ions, demonstrating its robustness.

Biofilms are implicated in approximately 80% of human microbial infections. They are also involved in the pathogenesis of foodborne diseases and contribute to antibiotic resistance (O’Toole et al., 2000). Therefore, the effectiveness of antimicrobials in eradicating biofilms must be evaluated. Endolysins are considered to be particularly effective at controlling bacterial biofilms (Gondil et al., 2020) Lys84 and LysSA52 have been shown to effectively remove 90 and 60% of *S. aureus* biofilms, respectively (Ning et al., 2021; Abdurahman et al., 2023). In this study, LysP70 significantly inhibited biofilm formation at 24 and 72 h and effectively removed preformed biofilms after 2 h. These results demonstrate that LysP70 can prevent biofilm formation and degrade established biofilms.

The expression levels of genes associated with biofilm formation were also evaluated. The quorum-sensing genes *agrA*, *agrB*, and *luxS,* which modulate bacterial signaling and biofilm synthesis (Karthikeyan et al., 2020), were significantly upregulated following LysP70 treatment, suggesting a bacterial response to external stress. Dos Santos (Rodrigues dos Santos et al., 2023) noted that piperine induced the upregulation of the *L. monocytogenes* genes *agrB*, *agrC*, and *agrD*, implying a defense mechanism against the impact of piperine on the bacterial cell membrane. The *flip*, *flgE*, *motB*, and *degU* genes, which are associated with flagellar structure, play a role in the regulation of flagellar assembly, modulation of bacterial adhesion, and motility, and consequently affect the capacity of bacteria to establish biofilms. The pronounced upregulation of the flagellar genes *flip*, *flgE*, *motB*, and *degU* after LysP70 treatment suggests a bacterial strategy to counteract adverse environmental conditions through augmented flagellar motility. This observation aligns with the findings of Byun et al. (2022), who reported substantial upregulation of *flaA* and *motB* within biofilms in response to phage treatment. Conversely, the downregulation of *flgE* and *degU* expression after 72 h of LysP70 exposure suggests a temporal reduction in flagellar function, potentially curtailing bacterial adhesion, and consequently affecting biofilm formation. Studies have demonstrated that the endolysins pEf 191 and pEf 51 can disrupt *Enterococcus faecalis* biofilms and inhibit the expression of genes associated with biofilm formation, including *sprE*, *ebpC*, *gelE*, and *esp* (Xiang et al., 2024a, 2024b).

The *lmo2504* gene, which encodes a cell wall-anchored protein critical for biofilm integrity, was significantly downregulated by LysP70 treatment, which likely compromised biofilm structure. Deletion of *lmo2504* in *L. monocytogenes* strain 3,119 results in a diminished capacity for biofilm formation, thereby substantiating the role of the gene in this process (Lourenço et al., 2013). The genes *actA* and *sigB*, identified as a key virulence factor and an environmental stress response regulator respectively, exert significant influence on biofilm formation in *L. monocytogenes* (Janež et al., 2021). In this study, LysP70 was applied to biofilms for 24 h. The marked upregulation of *actA* suggests a bacterial strategy to enhance survival under antimicrobial pressure through the increased expression of virulence factors. Upregulation of *sigB* reflects the adaptive mechanisms of bacteria in response to hostile environments. This is consistent with the literature, suggesting that bacteria sustain their viability under antimicrobial challenges by modulating the expression of virulence factors (Byun and Kim, 2023). Conversely, after 72 h of LysP70 exposure, the expression levels of *actA* and *sigB* were downregulated, which impeded biofilm development. Collectively, LysP70 exerted an inhibitory effect on biofilm formation by modulating the expression of various biofilm-associated genes. While the downregulation of certain genes may have directly impaired the structural and functional integrity of the bioepidermis, the upregulation of others may have been a part of the bacterial stress response and survival tactics in the presence of antimicrobial agents.

As a foodborne pathogen, *L. monocytogenes* poses significant health risks due to its propensity to contaminate food items. Therefore, it is imperative to identify effective bactericidal agents for use in the food industry. Several studies have demonstrated the efficacy of endolysins for mitigating foodborne bacteria in food products. The endolysins Ply500 and LysZ5, which are specific to *L. monocytogenes*, were effective in reducing bacterial contamination of lettuce and soy milk by 4 Log_10_ CFU/mL (Solanki et al., 2013; Zhang et al., 2012).

This study used the endolysin LysP70 to address artificially contaminated milk and lettuce samples. At refrigeration temperature of 4°C, milk exhibited a maximum bacterial reduction of 0.5 Log_10_ CFU/mL, whereas lettuce showed a 1.23 Log_10_ CFU/mL reduction, potentially due to the compositional influence of milk on lysis activity. The action of LysP70 action over a 4 h period at 30°C led to a maximum bacterial reduction of 1.9 Log_10_ CFU/mL in milk and 1.33 Log_10_ CFU/mL in lettuce, with a slightly superior efficacy in milk, likely due to the uneven distribution of the enzyme in lettuce, hindering adequate contact with bacteria. However, after 24 h, the bacterial counts in the experimental and control groups were comparable, likely owing to nutrient availability in the food matrix, which facilitated bacterial regrowth. Despite this, LysP70 demonstrated rapid and potent lytic capability within a short time frame, indicating that it may have application value in specific scenarios where short-term antibacterial effects are required. Subsequent studies need to further verify its continuous effect in complex food systems.

LysP70 demonstrated potent lytic activity against *Listeria* species, exhibiting remarkable stability, a broad host range, and significant inhibitory and eradication effects on biofilms. Furthermore, it had a pronounced effect on the transcriptional levels of genes associated with biofilm formation, underscoring its multifaceted antibacterial potential. The preliminary application efficacy of this LysP70 was assessed in both milk and lettuce, revealing its capacity to significantly diminish the population of *Listeria monocytogenes* in food within a short timeframe. However, the related application potential still needs to be confirmed through more systematic verification of food matrices and long-term stability studies. This study provides a strong foundation for the future development and application of endolysins in both the food safety and medical fields, paving the way for innovative solutions to combat bacterial infections and biofilm-related challenges.

## Data Availability

The original contributions presented in the study are included in the article/Supplementary material, further inquiries can be directed to the corresponding author/s.

## Footnotes

^1^ https://web.expasy.org/protparam/

^2^ https://www.ncbi.nlm.nih.gov/Structure/cdd/wrpsb.cgi

^3^ https://swissmodel.expasy.org/interactive
