# Supplementary material for: Antibacterial activity of endolysin LysP70 from Listeria monocytogenes phage
Source: Front Microbiol. 2025 Jul 15;16:1566041. doi: 10.3389/fmicb.2025.1566041 (PMC12303923; doi:10.3389/fmicb.2025.1566041)
Supplement: Supplementary file 1 [file Data_Sheet_1.docx]

**Appendix 1 Molecular weight isoelectric point analysis of LysP70 protein**

| Item | Results |
| --- | --- |
| Number of amino acids | 315 |
| Molecular of weight | 34226.14 |
| Theoretical of pI | 9.86 |
| Total number of negatively charged residues (Asp+Glu) | 22 |
| Total number of positively charged residues (Arg+Lys) | 47 |
| Estimation of half-life | The N-terminal of the sequence considered is M (Met).  30 hours (mammalian reticulocytes, in vitro).>20 hours (yeast, in vivo).>10 hours (Escherichia coli, in vivo). |
| Instability index | The instability index (II) is computed to be 20.39  This classifies the protein as stable. |
| Aliphatic index | 64.44 |
| Grand average of hydropathicity | -0.595 |


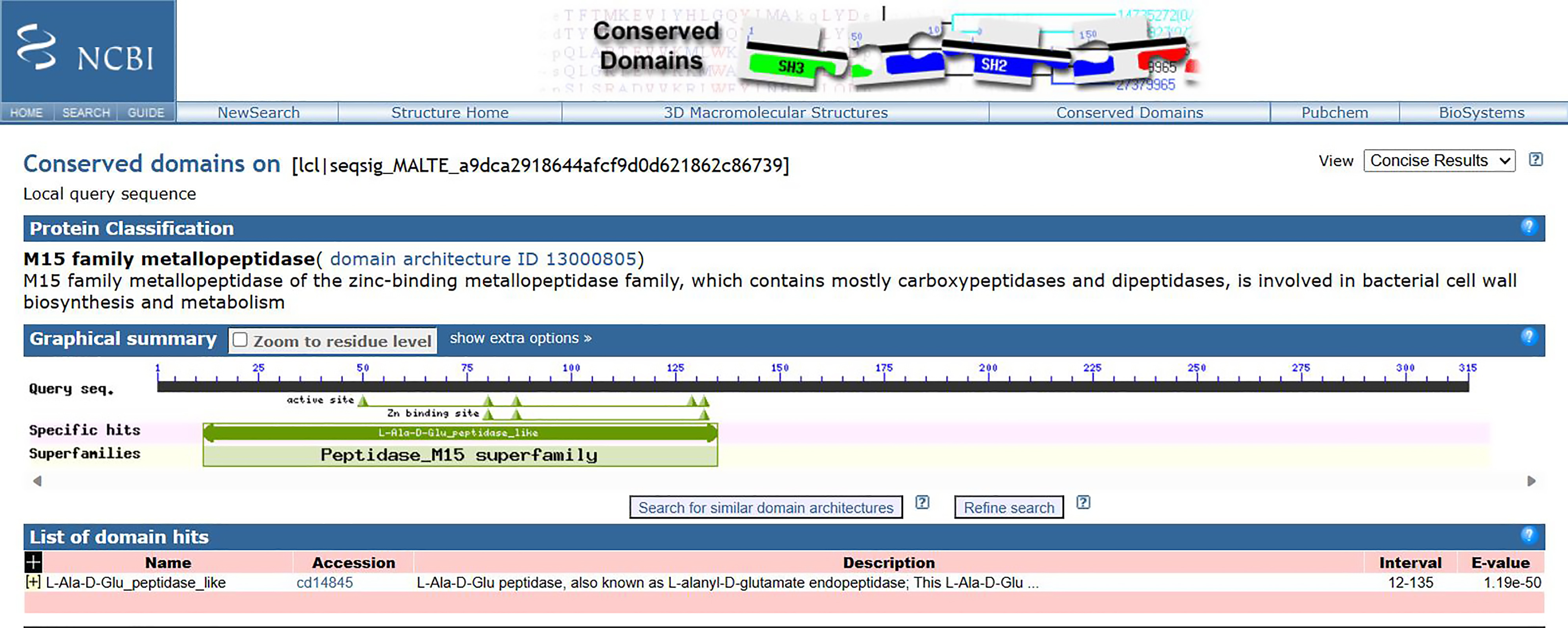


**Appendix 2 Prediction of the conserved domain of LysP70 protein**


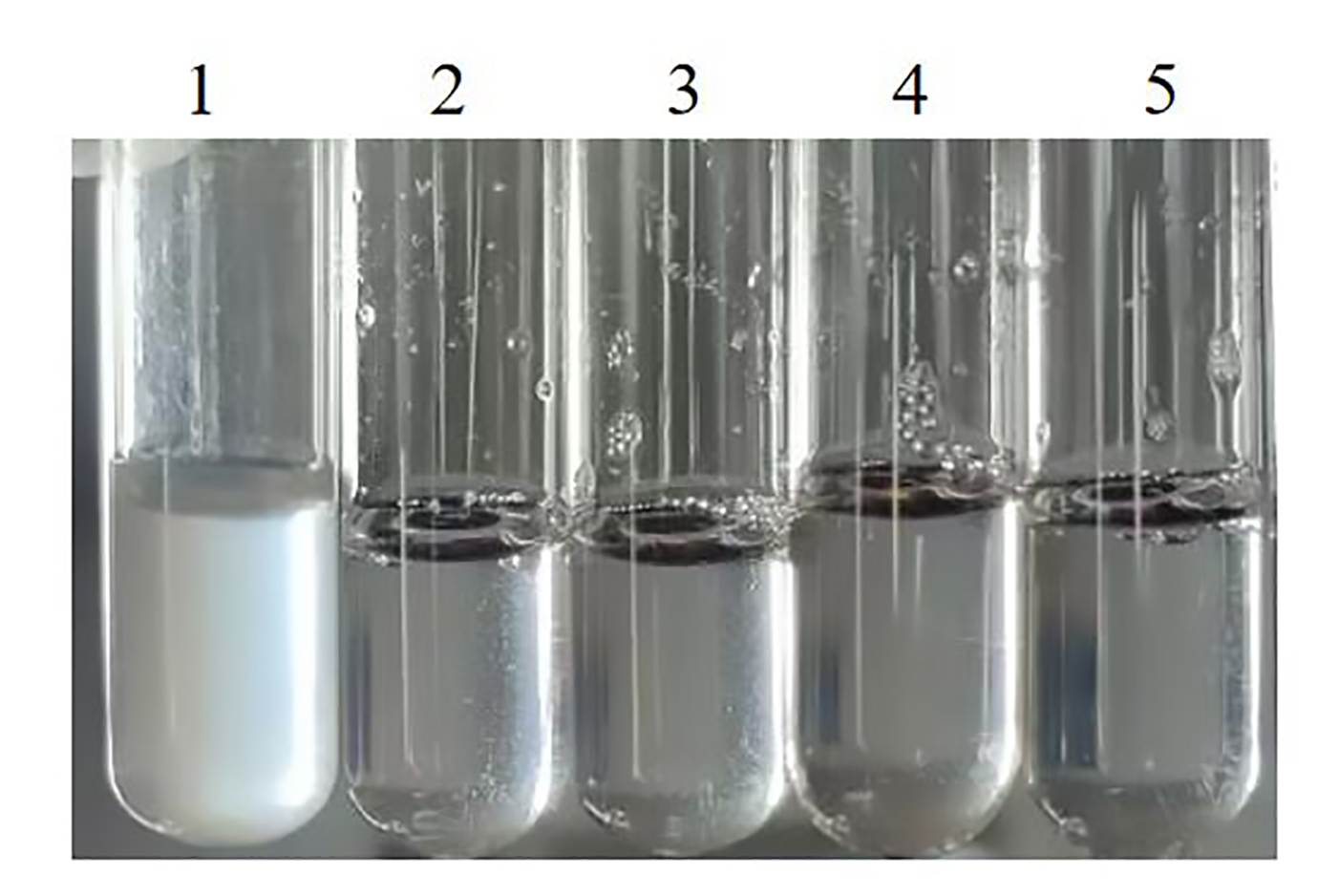


**Appendix 3 Lytic activity of endolysin LysP70 in liquid environment**

Note: 1: Control; 2: 400 µg/mL; 3: 200 µg/mL; 4: 100 µg/mL; 5: 50 µg/mL
